# Supplementary material for: High-dimensional neural network potentials for accurate vibrational frequencies: the formic acid dimer benchmark
Source: Phys Chem Chem Phys. 2022 Nov 24;24(48):29381–92. doi: 10.1039/d2cp03893e (PMC9749085; doi:10.1039/d2cp03893e)
Supplement: CP-024-D2CP03893E-s001 [file CP-024-D2CP03893E-s001.pdf]

Electronic Supplementary Material (ESI) for Physical Chemistry Chemical Physics.
This journal is © the Owner Societies 2022

Supporting information for:

High-dimensional neural network potentials for accurate vibrational frequencies: The formic acid dimer benchmark

Dilshana Shanavas Rasheeda,*[a] Alberto Martín Santa Daría,[b] Benjamin Schröder,[c] Edit Mátyus[b] and Jörg Behler[a,‡]

[a] Universität Göttingen, Institut für Physikalische Chemie, Theoretische Chemie, Tammannstraße 6, 37077 Göttingen, Germany.
[b] ELTE, Eötvös Loránd University, Institute of Chemistry, Pázmány Péter sétány 1/A, 1117 Budapest, Hungary
[c] Universität Göttingen, Institut für Physikalische Chemie, Tammannstraße 6, 37077 Göttingen, Germany.
‡ Present address: Lehrstuhl für Theoretische Chemie II, Ruhr-Universität Bochum, 44780 Bochum, Germany, and Atomistic Simulations, Research Center Chemical Sciences and Sustainability, Research Alliance Ruhr
* E-mail: dilshana.rasheeda@chemie.uni-goettingen.de

A Construction of the HDNNPs

The common settings for the construction of high-dimensional neural network potentials (HDNNPs) is given in Table S1. For different iterations of the HDNNPs, different atom centred symmetry functions (ACSFs) were employed. ACSFs are used to represent the atomic environments and ensure translational and rotational invariance. For the construction of each of the iterations of the HD-NPPs, additional structures are added (see main manuscript for details). As a result, the atomic environment also has to be redefined within each iteration. The parameters[1] of the ACSFs used for constructing the various HDNNPs are given in Tables S2, S3, S4, and S5. The cutoff radius, as needed for the definition of the ACSFs,[1] is 14.901 Bohr for HDNNP1 and 15.0 Bohr for HDNNP2 as well as HDNNP3, i.e. the final FAD-HDNNP intended for production use.

Table S1 RuNNer settings for HDNNPs

Keyword	Settings
nn_type_short	1
random_number_type	5
global_activation_short	t t l
cutoff_type	1
use_short_nn	
global_hidden_layers_short	2
scale_symmetry_functions	
center_symmetry_functions	

Table S2 Radial ACSF parameters η for HDNNP1

element pair	$\eta\,[\mathrm{Bohr}^{-2}]$
H-H	0, 0.003320, 0.007822, 0.014296, 0.024263, 0.040982, 0.072561, 0.144102
O-O	0, 0.002331, 0.005208, 0.008869, 0.013680, 0.020235, 0.029556, 0.043520
C-C	0, 0.000964, 0.002013, 0.003161, 0.004425, 0.005824
H-C	0, 0.003763, 0.009087, 0.017202, 0.030743, 0.056242, 0.113944, 0.295433
O-C	0, 0.003648, 0.008752, 0.016415, 0.028926, 0.051756, 0.100815, 0.240433
H-O	0, 0.003910, 0.009520, 0.018245, 0.033218, 0.062638, 0.134133, 0.395239

Table S3 Radial ACSF parameters η for HDNNP2 and HDNNP2a

element pair	$\eta\,[\mathrm{Bohr}^{-2}]$
H-H	0, 0.004000, 0.009000, 0.016000, 0.028000, 0.049000, 0.094000, 0.215000
O-O	0, 0.003000, 0.006000, 0.010000, 0.015000, 0.022000, 0.032000, 0.048000
C-C	0, 0.003747, 0.009066, 0.017212, 0.030893, 0.056909
H-C	0, 0.004000, 0.009000, 0.018000, 0.031000, 0.056000, 0.114000, 0.296000
O-C	0, 0.004000, 0.009000, 0.017000, 0.029000, 0.052000, 0.101000, 0.241000
H-O	0, 0.004000, 0.010000, 0.019000, 0.033000, 0.063000, 0.134000, 0.395000

Table S4 Radial ACSF parameters η for HDNNP3 (=FAD-HDNNP)

element pair	η [Bohr^{-2}]
H-H	0, 0.004000, 0.009000, 0.016000, 0.028000, 0.049000, 0.094000, 0.215000
O-O	0, 0.003000, 0.006000, 0.010000, 0.015000, 0.022000, 0.032000, 0.048000
C-C	0, 0.003747, 0.009066, 0.017212, 0.030893, 0.056909
H-C	0, 0.004000, 0.009000, 0.018000, 0.031000, 0.056000, 0.114000, 0.296000
O-C	0, 0.004000, 0.009000, 0.017000, 0.029000, 0.052000, 0.101000, 0.241000
H-O	0, 0.004000, 0.010000, 0.019000, 0.035000, 0.067000, 0.149000, 0.486000

Table S5 Angular ACSF parameters η, ζ, and λ employed for all element combinations and HDNNPs

No.	η [Bohr^{-2}]	ζ	λ
1	0.0	1.0	1.0
2	0.0	2.0	1.0
3	0.0	4.0	1.0
4	0.0	16.0	1.0
5	0.0	1.0	-1.0
6	0.0	2.0	-1.0
7	0.0	4.0	-1.0
8	0.0	16.0	-1.0

B Transition state of the double proton transfer

The geometries of the double-proton transfer transtition state (TS) were optimized on the HDNNPs as well as the QB16 PES[2] and the corresponding barrier height $\Delta E^{\ddagger}$ evaluated. Starting from the TS structure of FAD-HDNNP the geometry was also optimized at the reference *ab initio* level, i.e. fc-CCSD(T)-F12a/haTZ with *tight* optimization settings. The latter calculations slightly improve on the previous results of Qu and Bowman[2] who obtained the barrier height by performing fc-CCSD(T)-F12a/haTZ single point calculations on fc-CCSD(T)-F12a/haDZ geometries. Key geometrical parameters and the resulting barrier heights are compiled in Table S6.

Table S6 Geometrical parameters of the transition state structure for double proton transfer in the formic acid dimer optimized at the reference *ab initio* level of theory and determined for different PESs. Bond lengths are provided in Ångströms and angles in degrees. Additionally, the barrier-height $\Delta E^{\ddagger}$ is quoted in cm^{-1}. HDNNP3 corresponds to the final FAD-HDNNP for spectroscopic use.

Parameter	*Ab initio*[a]	QB16[b]	HDNNP1	HDNNP2	HDNNP3
r(O–H)	1.2033	1.2049	1.2009	1.2013	1.2016
r(C–H)	1.0923	1.0923	1.0905	1.0922	1.0919
r(C–O)	1.2595	1.2595	1.2598	1.2595	1.2594
r(O···O)	2.4059	2.4091	2.4011	2.4020	2.4026
∠O=C–O	126.63	126.61	126.72	126.84	126.68
∠O=C–H	116.68	116.70	116.64	116.58	116.66
∠C–O–H	115.38	115.42	115.21	115.38	115.39
∠O–H–O	177.39	177.44	177.13	177.60	177.47
$\Delta E^{\ddagger}$	2854	2848	2861	2858	2866

[a] CCSD(T)-F12a/haTZ using *tight* settings.
[b] Qu and Bowman[2]

C Quartic force fields

The parameters of the quartic force field (QFF) for the formic acid dimer (FAD) were obtained by numerical differentiation. To this end, diagonalization of the (mass-weighted) numerical hessian yielded the harmonic frequencies ω_i and normal coordinate displacement vectors. The cubic and quartic force constants ϕ_{ijk} and ϕ_{ijkl} (see manuscript and Ref. 3 for a definition) are calculated by standard finite difference formulas with up to 5 points per coordinate. A uniform value of 0.01 was chosen for the step size in terms of the dimensionless normal coordinates. Due to symmetry a large number of the ϕ_{ijk} and ϕ_{ijkl} vanish, i.e. only those are different from zero for which the direct product of the irreducible representations of the involved normal coordinates is totally symmetric.

Parameters of the QFF (equilibrium geometry, normal coordinates and force constants) were deposited in GRO.data (doi:10.25625/ZDGKYA).[4] The equilibrium geometry (see also Tables S7 and S8) is provided in a formatted ASCII file named FAD.XYZ and the force constants of the QFF in a file named QFF. The normal coordinate vectors that determine the Coriolis ζ_{ij}^{α} needed to reproduce the VPT2 results provided in the main manuscript can be found in an file named QCOORDS. In these files only non-vanishing combinations of $\{i, j, k, l\}$ with $i \leq j \leq k \leq l$ are quoted. Note that for standard VPT2 only the semi-diagonal QFF is relevant,[5] i.e. only those quartic force constants of type ϕ_{iiii} and ϕ_{iijj} contribute to the transition frequencies. The corresponding files for the reference *ab initio* VPT2 calculation are also provided. A comparison of the QFF force constants obtained with the HDNNPs and the reference *ab initio* values is provided in Figure S1.

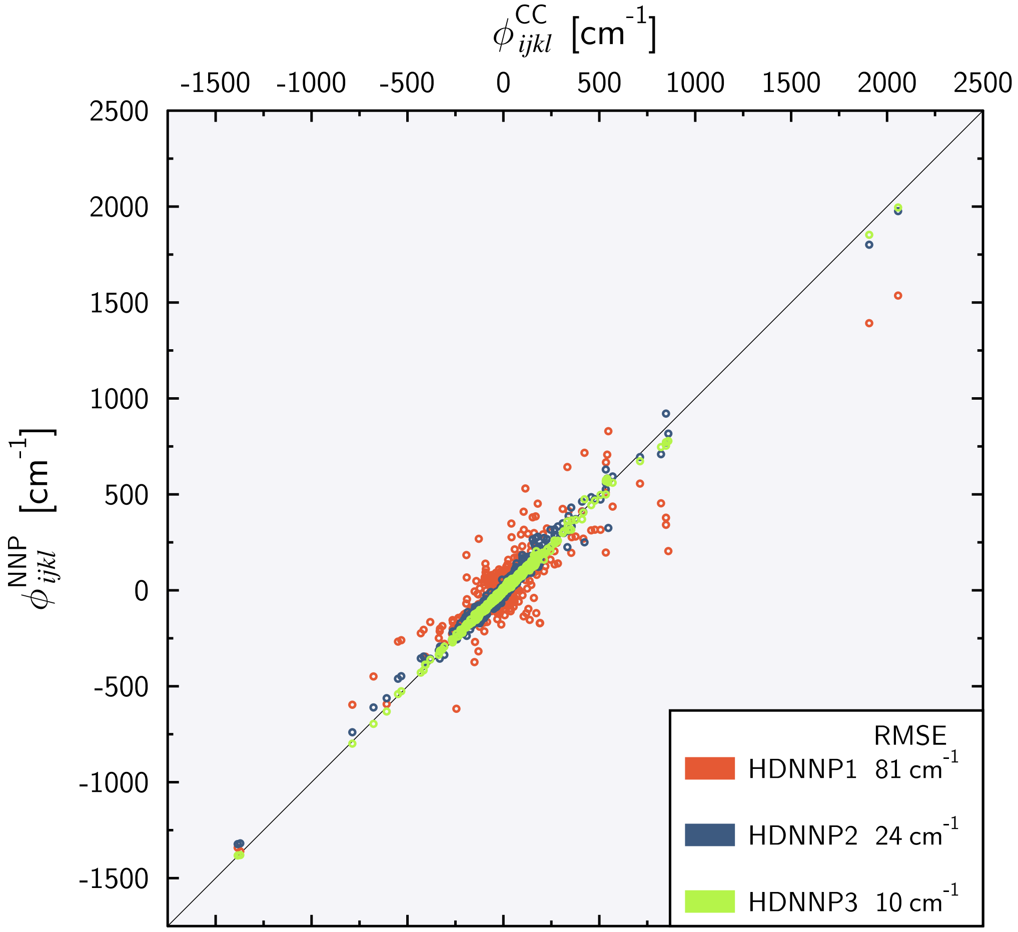

Fig. S1 Comparison of semi-diagonal quartic force field parameters ϕ_{ijkl} [cm^{-1}] obtained with the reference *ab initio* method (CC) and from different HDNNPs.

Table S7 Cartesian coordinates [Å] of the FAD minimum structure obtained with FAD-HDNNP.

Atom	x	y	z
H	0.2631043230	0.0000000000	2.9819981733
H	−0.2631043230	0.0000000000	−2.9819981733
H	−1.1172113912	0.0000000000	0.5090506679
H	1.1172113912	0.0000000000	−0.5090506679
O	1.1554707969	0.0000000000	1.1679005999
O	−1.1554707969	0.0000000000	−1.1679005999
O	−1.0747774476	0.0000000000	1.5017795982
O	1.0747774476	0.0000000000	−1.5017795982
C	0.1767682702	0.0000000000	1.8926694202
C	−0.1767682702	0.0000000000	−1.8926694202

Table S8 Cartesian coordinates [Å] of the FAD minimum structure obtained at the fc-CCSD(T)-F12a/haTZ level of theory (*tight* settings).

Atom	x	y	z
H	0.2611070499	0.0000000000	−2.9847855230
C	0.1762661637	0.0000000000	−1.8951307992
O	1.1552690117	0.0000000000	−1.1711296421
O	−1.0752968564	0.0000000000	−1.5035144553
H	−1.1166848159	0.0000000000	−0.5111726141
H	−0.2611070499	0.0000000000	2.9847855230
C	−0.1762661637	0.0000000000	1.8951307992
O	−1.1552690117	0.0000000000	1.1711296421
O	1.0752968564	0.0000000000	1.5035144553
H	1.1166848159	0.0000000000	0.5111726141

Notes and references

1 J. Behler, *J. Chem. Phys.*, 2011, **134**, 074106.

2 C. Qu and J. M. Bowman, *Phys. Chem. Chem. Phys.*, 2016, **18**, 24835–24840.

3 D. A. Clabo, W. D. Allen, R. B. Remington, Y. Yamaguchi and H. F. Schaefer, *Chem. Phys.*, 1988, **123**, 187–239.

4 D. S. Rasheeda, A. M. Santa Daría, B. Schröder, E. Mátyus and J. Behler, *Replication Data for: High-dimensional neural network potentials for accurate vibrational frequencies: The formic acid dimer benchmark*, GRO.data, 2022, `https://doi.org/10.25625/ZDGKYA`.

5 D. Papoušek and M. R. Aliev, *Molecular Vibrational-rotational Spectra: Theory and Applications of High Resolution Infrared, Microwave and Raman Spectroscopy of Polyatomic Molecules*, Elsevier Science Ltd, 1982.
